# HLA-B*35 as a new marker for susceptibility to human T-cell lymphotropic virus type 1 (HTLV-1) Associated Myelopathy/Tropical Spastic Paraparesis (HAM/TSP) in patients living in Argentina

**DOI:** 10.1186/s12977-020-00536-y

**Published:** 2020-09-03

**Authors:** Paula Benencio, Sindy A. Fraile Gonzalez, Nicolás Ducasa, Kimberly Page, Carolina A. Berini, Mirna M. Biglione

**Affiliations:** 1 CONICET- Universidad de Buenos Aires, Instituto de Investigaciones Biomédicas en Retrovirus y SIDA (INBIRS), Paraguay 2155, C1121ABG Ciudad Autónoma de Buenos Aires, Argentina; 2 Department of Epidemiology and Biostatistics, University of California San Francisco, San Francisco, CA USA; 3 Present Address: The University of New Mexico, Internal Medicine, University of New Mexico, Albuquerque, NM USA

**Keywords:** HTLV-1, HAM/TSP, ATLL, PVL, HLA, ARGENTINA

## Abstract

**Background:**

Human T-cell lymphotropic virus type 1 (HTLV-1) is the etiological agent of HTLV associated myelopathy/ Tropical Spastic Paraparesis (HAM/TSP) and Adult T cell leukemia/lymphoma (ATLL), in around 2–5% of the infected individuals. Host genetic background might play a role in disease progression. Several previous studies across many countries report HLA haplotype to be one such factor. Here, we sequenced HLA-A, -B and -C of 66 individuals by Sequence-Based Typing (SBT), and compared the frequency of different alleles among ATLL patients, HAM/TSP patients, asymptomatic carriers and non-infected individuals living in Argentina.

**Results:**

The frequency of HLA-A, -B and -C alleles largely matched that of the general population in Argentina. We identified HLA-A*02, HLA-B*35 and HLA-C*07 as associated to protection from ATLL (*p* = 0.031), susceptibility to HAM/TSP (*p* < 0.001) and susceptibility to ATLL (*p* = 0.017), respectively. We also found a strong correlation between high proviral load (PVL) and disease (*p* = 0.008), but were unable to identify any particular allele associated with high or low PVL.

**Conclusions:**

We have found HLA-A*02, HLA-B*35 and HLA-C*07 to be associated to protection from ATLL (HLA-A*02) and susceptibility to HAM/TSP (HLA-B*35) or to ATLL (HLA-C*07), respectively. Whereas HLA-A*02 protection from ATLL has already been extensively described in other regions of the world, this is the first report that links HLA-B*35 and an increased susceptibility to HAM/TSP. As for HLA-C*07 it has previously been associated to susceptibility to HAM/TSP in other countries but in our population it has been linked to ATLL.

## Background

Human T-cell lymphotropic virus type 1 (HTLV-1) was the first human retrovirus to be discovered and is the etiological agent of Adult T Cell Leukemia/Lymphoma (ATLL), a progressive neurological disease called HTLV-1 Associated Myelopathy/Tropical Spastic Paraparesis (HAM/TSP) [1, 2], Uveitis, a severity factor for Bronchiectasis and other diseases [3]. In Argentina, the Northwestern region (Salta and Jujuy provinces) is considered endemic for HTLV-1 infection [4].

The vast majority of HTLV-1-infected individuals are asymptomatic and around 1–2% of them will develop ATLL and less than 5%, HAM/TSP. Although the risk factors causing different HTLV-1 associated diseases are not fully understood, their pathogenesis is thought to be in part due to proviral load (PVL) and/or the host genetic factors [5–7].

The target cells of HTLV-1 are CD4^+^ T cells, and to a lesser extent CD8 + T cells, B cells, monocytes, macrophages and dendritic cells [7]. The maintenance of HTLV-1 infection occurs mostly by clonal expansion of infected cells. In the case of ATLL, the expression of Tax protein in some of these clones with accumulated genomic abnormalities could help develop a pre-leukemic state in some individuals. The malignantly transformed HTLV-1 infected cells very often suppress Tax expression in favor of HTLV-1 basic leucine zipper factor (HBZ) expression, a negative regulator of Tax that is believed to aid immune evasion of the infected cells and further assist cancerous transformation [8]. On the other hand, the increased number of HTLV-1 infected T-cells may also cause imbalance of the immune system, resulting in immune dysfunction or inflammatory diseases like myelopathy and uveitis [9]. In this context, HAM/TSP pathogenesis is a hyperactive immune response induced by HTLV-1 infection that produces chronic inflammation in the central nervous system (CNS) with slowly progressive evolution. It is characterized by the production of elevated levels of proinflammatory cytokines, including IFN-ɣ and TNF, and by HTLV-1-specific CD8^+^ T cells in peripheral blood and spinal cord lesions [10–12].

Human Leukocyte Antigen (HLA) class I genes have been associated with susceptibility to disease in many human infections and it is among the host genetic factors that could be related to manifestation of ATLL and HAM/TSP [13, 14]. A unifying theory is that HLA alleles associated with ATLL show a limited recognition of HTLV-1 Tax peptide anchor motifs and epitopes capable of generating anti-HTLV-1 Tax CD8^+^ T cells while for HAM/TSP they induce strong cytotoxic T-lymphocyte (CTL) responses against the viral oncoprotein Tax [15, 16].

Specific HLA alleles have been linked to protection from developing these pathologies, whereas other HLA alleles have been correlated with an increased risk of developing them (Table 1). Some studies reported the HLA-A*02 allele to have a protective role both in ATLL and HAM/TSP disease in Jamaica, Brazil, Japan and Peru [16–21]. The same was the case for allele HLA-Cw*08 in Japan and Iran [16, 20, 22]. HLA-A*26 and A*54 were associated with susceptibility to ATLL and HAM/TSP, respectively, in Japan, whereas A*36 was associated with susceptibility to ATLL in Jamaica [17, 23]. In contrast, HLA-B*5401 has been associated with an increased susceptibility to HAM/TSP in Japan and Iran [16, 24]. Allele C*07, on the other hand, has been associated to susceptibility to disease in Brazil, only in patients who did not possess A*02 [25].

**Table 1:** Alleles previously associated with either protection or susceptibility to HTLV-1 associated diseases

Country/Continent (Reference)	N, Population	Protective allele	Detrimental allele
Africa [52]	N = 45, Ac N = 49, ATLL N = 51, NII	–	HLA-A*36 HLA-B*18
Brazil [18, 19]	N = 71, HTLV-1 + N = 188, NII	HLA-A*02 (HAM)	HLA-A*26 (ATLL)
	N = 53, Ac N = 55, HAM	HLA-A*02	–
Spain [14]	N = 40, Ac N = 20, HAM	–	HLA-B*5401 absent HLA-B*07
Iran [22, 24, 49]	N = 74, Ac N = 58, HAM	–	HLA-B*5401
	Iranian N = 74, Ac N = 58, HAM Kagoshima N = 184, Ac N = 222, HAM	HLA-Cw*08	–
	N = 20, HAM N = 30, Ac	No association: HLA-A*02 HLA-A*24 HLA-Cw*08	–
Jamaica [17, 28]	N = 45, Ac N = 25, ATLL	HLA-A*02 HLA-A*33	HLA-A*03 HLA-A*36
	N = 56, ATLL N = 59, HAM	HLA-A*03	HLA-B*15 HLA-B*53
Japan [16, 20, 23, 53]	N = 34, ATLL N = 55, HAM N = 241, AC	–	HLA-A*26 (ATLL) HLA-B*61 (ATLL) HLA-B*07 (HAM) HLA-Cw*07 (HAM)
	N = 175, NII N = 152, Ac N = 124, ATLL N = 148, HAM	–	HLA-A*26 HLA-B*4002 HLA-B*4006 HLA-B*4801
	N = 233, HAM N = 202, NII	HLA-A*02 HLA-Cw*08	HLA-B*5401
	N = 201, Ac N = 232, HAM	HLA-A*0201 HLA-Cw*0801	HLA-B*5401
Peru [21]	N = 71, HAM N = 94, Ac	HLA-A*02	–

*Ac* asymptomatic carriers, *HAM/TSP* HTLV-1 associated myelopathy/Tropical Spastic Paraparesis, *ATLL* Adult T cell leukemia/lymphoma, *NII* Non-infected Individuals, *HLA* Human leukocyte antigen

Another perspective suggests that greater HLA diversity conveys selective advantage against disease because the immune response is elicited by a greater variety of antigens as described for human immunodeficiency virus (HIV) and acquired immunodeficiency syndrome (AIDS) [26]. Since each HLA allele exposes a different set of amino acids in their peptide cleft, they will each be able to present peptides with different specificities. This results in a heterogeneous capacity to activate T CD8^+^ lymphocytes to target the infected cells. Carrying mismatched alleles for each HLA gene thus confers the ability to present a wider range of peptides, and to then be more likely to activate cytotoxic T cells to eliminate infected cells. It has been reported that, in HIV infection, HLA class I heterozygotes progress more slowly to AIDS than do homozygotes and that the viral load is significantly lower due to rare HLA class I alleles [27]. In relation to HTLV-1 infection, Goedert et al. showed that HLA class I diversity reduces the risk of ATLL presumably by limiting the proliferation of HTLV-1 infected cells in vivo and therefore decreasing the possibilities of developing the disease [28].

The aim of this study was to identify HLA class I alleles associated to protection or susceptibility to disease and to analyze its possible association with proviral load in individuals infected with HTLV-1 living in Argentina.

## Methods

Peripheral blood samples were obtained from a total of 66 individuals, of which 52 presented HTLV-1 infection (9 ATLL, 22 HAM/TSP, 21 asymptomatic carriers (AC)) and 14 non-infected individuals (NII). All of them attended our Institute seeking HTLV-1/2 diagnosis between 2003 and 2018.

The protocol was reviewed and approved by the Institutional Review Board as well as by the External Ethical Committee (NEXO AC IRB#0005349, protocol #1563). An informed consent was obtained from all individuals. The diagnosis of ATLL and HAM/TSP was performed in accordance with Tsukasaki and Osame criteria, respectively [29]. Peripheral blood mononuclear cells (PBMCs) were isolated from EDTA-treated blood samples by a Ficoll-Hypaque density gradient (Ficoll Paque Plus, Sigma Aldrich, Saint Louis, USA) and DNA was extracted using a commercial kit (ADN PuriPrep-S kit, Inbio Highway, Tandil, Argentina). After serological screening, HTLV-1 infection was confirmed by an in-house nested polymerase chain reaction (n-PCR) as described elsewhere [30]. Absolute quantitation of PVL was performed by real-time SYBR Green PCR, using an ABI Prism 7500 Prism System (Applied Biosystems, USA) as previously described [31, 32].

HLA class I characterization was performed by sequence based typing (SBT). HLA-A exons 2 and 3 were amplified together while HLA-B/C exons 2 and 3 were amplified separately, as described elsewhere [33].

Amplicons were sequenced using the Big Dye Terminator sequencing kit (Applied Biosystems, USA) on a 3500xL Genetic Analyzer AB/HITACHI according to the manufacturer’s instructions.

For the calculation of allele frequencies, we counted each individual allele for each locus across all samples. In the cases where an individual resulted homozygous for a locus, this situation was equivalent to a count of 2 for the corresponding allele.

For the analysis of homozygosis/ heterozygosis, we excluded from the total count those individuals whose haplotypes could not be typed, or were partially elucidated.

Data analysis was performed using the Kruskal–Wallis non-parametric method; when two groups were compared the Chi^2^ test or Exact Fisher test, and one-way ANOVA were used. Epidat (version 4.2) and GraphPad Prism (version 6.03) software was applied and significant differences were defined as *p* < 0.05.

## Results

We analyzed a total of in 66 individuals living in Argentina of whom 16 were born in other South American countries such as Paraguay (6.1%, n = 4) and Peru (18.2%, n = 12). Among the 66 samples analyzed, there was no significant difference in biological sex (*p* = 0.410, Table 2) between asymptomatics and the group with HTLV-1 associated pathologies. On the other hand, there was a significant difference in age (*p* = 0.002, mean age = 41.05, mean age NII = 32.27, mean age AC = 38.6, mean age HAM/TSP = 46.57, mean age ATLL = 44.33, Table 2) when analyzing the same groups. Table 2 presents the demographic characteristics of all studied individuals. None of the Argentine individuals reported being born nor have they been derived from endemic areas for HTLV-1 infection in the country.

**Table 2:** Demographic characteristics of the studied population

Code	Population	City/ Country of derivation	Birthplace	Biological sex	Age (years)	PVL
132,253	NII	Buenos Aires Province	Buenos Aires Province	M	31	NA
132,254	HAM	Buenos Aires Province	Buenos Aires Province	M	50	1.35
153,302	AC	Buenos Aires City	Buenos Aires City	F	47	ND
154,545	NII	Buenos Aires City	Buenos Aires City	F	22	NA
201,437	AC	Buenos Aires City	Buenos Aires Province	M	31	0.39
203,568	AC	Buenos Aires City	Perú	F	36	2.91
203,835	AC	Buenos Aires City	Buenos Aires Province	M	18	0,68
325,784	NII	Buenos Aires Province	Peru	F	36	NA
327,362	AC	Buenos Aires Province	Buenos Aires Province	M	61	5.3
327,557	AC	Buenos Aires Province	Buenos Aires Province	F	42	11.83
2,013,082	NII	Buenos Aires Province	Peru	F	41	NA
2,013,100	HAM	Buenos Aires City	Buenos Aires Province	M	49	4.03
2,013,102	AC	Buenos Aires City	Buenos Aires Province	F	36	0.29
2,014,059	ATLL	Buenos Aires City	Entre Ríos	F	62	ND
2,014,090	NII	Buenos Aires City	Buenos Aires City	F	38	NA
2,014,101	AC	Buenos Aires City	Buenos Aires City	M	32	0.58
2,014,102	AC	Buenos Aires City	Paraguay	M	56	1.29
2,014,150	AC	Buenos Aires Province	Peru	M	34	4.61
2,014,151 = 2,016,111	HAM	Buenos Aires Province	Peru	F	61	12.86
2,014,160	AC	Buenos Aires City	Buenos Aires City	F	NA	0,57
2,014,161	NII	Buenos Aires City	Peru	M	NA	NA
2,015,014	AC	Buenos Aires City	Paraguay	M	34	1.12
2,015,024	AC	Buenos Aires City	Peru	F	39	0,09
2,015,025	AC	Buenos Aires City	Peru	M	43	2.51
2,015,038	NII	Buenos Aires City	Paraguay	F	38	NA
2,015,059	NII	Santa Fe	Santa Fe	F	23	NA
2,015,062	NII	Santa Fe	Santa Fe	M	20	NA
2,015,074 = 2,017,009	AC	Buenos Aires Province	Peru	F	39	10.77
2,015,082	NII	Buenos Aires City	Buenos Aires Province	F	49	NA
2,015,090	AC	Buenos Aires City	Paraguay	F	27	0.79
2,015,106	HAM	Tucumán	Perú	F	41	2.00
2,015,107	ATLL	Buenos Aires City	Buenos Aires Province	M	18	9.87
2,015,116	NII	Buenos Aires City	Misiones	M	30	NA
2,016,001	NII	Buenos Aires City	Buenos Aires City	F	27	NA
2,016,002	AC	Buenos Aires City	Buenos Aires City	M	28	0.92
2,016,010	HAM	Santa Fe	Santa Fe	F	45	10.3
2,016,049	AC	Buenos Aires City	Peru	F	47	0.19
2,016,050	AC	Buenos Aires City	Peru	M	52	1.46
2,016,065	AC	Santa Fe	Santa Fe	M	48	9.24
2,016,066	AC	Santa Fe	Santa Fe	M	22	0.11
20,110,055 = 20,110,063	ATLL	Buenos Aires City	Tucumán	F	66	40.2
20,110,057	NII	Buenos Aires City	Tucumán	F	NA	NA
20,110,059	NII	Buenos Aires City	Tucumán	M	NA	NA
44,971	HAM	Buenos Aires City	NA	F	25	3.12
45,674	HAM	NA	NA	F	67	1.67
46,793	HAM	Buenos Aires Province	NA	NA	NA	ND
49,067	HAM	NA	NA	M	55	15.53
138,398	HAM	NA	NA	F	56	29.54
146,570	HAM	Buenos Aires Province	NA	F	51	4.78
170,313	ATLL	Buenos Aires City	NA	F	29	ND
174,908	HAM	Buenos Aires City	NA	M	19	0.12
180,405	HAM	Buenos Aires City	NA	F	71	10.45
192,816	HAM	Buenos Aires City	NA	F	37	ND
195,086	ATLL	Buenos Aires City	NA	F	48	1.30
198,544	ATLL	Buenos Aires City	NA	F	67	12.49
209,803	HAM	Buenos Aires City	NA	F	27	3.12
228,185	ATLL	Buenos Aires City	NA	M	53	33.98
244,886	ATLL	Santa Fe	NA	M	31	ND
248,684	HAM	Buenos Aires City	NA	M	65	5.39
255,404	HAM	Buenos Aires City	NA	M	31	ND
20,110,028	HAM	Buenos Aires City	NA	F	52	8.57
2,012,028	HAM	Buenos Aires City	NA	M	52	35.10
2,012,135	ATLL	Buenos Aires City	NA	M	25	0,34
20,110,001	HAM	Buenos Aires City	NA	F	43	0.71
2,014,057	HAM	Buenos Aires City	NA	M	41	13.09
2,014,063	HAM	Tucumán	NA	F	40	1.01

*NII* Non-infected Individual, *AC* Asymptomatic Carrier, *HAM* HTLV-1 associated myelopathy, *ATLL* adult T cell leukemia/lymphoma, *F* Female, *M* Male, *PVL* proviral load, *NA* not available, *ND* not determined

Out of the 66 samples included in the study, a total of 53 were typed for HLA-A (13 NII, 17 AC, 7 ATLL, 16 HAM/TSP), 61 for HLA-B (14 NII, 19 AC, 7 ATLL, 21 HAM/TSP) and 38 of them were analyzed for HLA-C (14 NII, 18 AC, 3 ATLL, 3 HAM/TSP). There were also a total of 14 samples for which only one of the alleles for a specific HLA I loci could be identified. Therefore, a total of 103 alleles could be typed for HLA-A, 112 for HLA-B and 74 for HLA-C.

Overall, A*02 (36.89%), B*35 (25.00%) and C*07 (33.78%) were the most frequent in the population studied. Tables 3, 4 and 5 shows the frequencies of individual alleles in all the studied individuals.

**Table 3:** Number and frequency of every HLA-A allele found in our population for each group

Allele	Population				Overall	Allele frequency
	NII	AC	ATLL	HAM		
HLA-A*01	4	0	0	0	4	0.039
HLA-A*02	7	17	2	12	38	0.369
HLA-A*03	2	1	0	0	3	0.029
HLA-A*11	0	0	0	2	2	0.019
HLA-A*23	0	0	1	0	1	0.010
HLA-A*24	4	4	1	0	9	0.087
HLA-A*25	0	1	0	0	1	0.010
HLA-A*26	0	1	0	0	1	0.010
HLA-A*29	2	0	0	4	6	0.058
HLA-A*31	1	5	4	7	17	0.165
HLA-A*32	0	0	2	0	2	0.019
HLA-A*33	1	3	1	6	11	0.107
HLA-A*68	3	1	2	0	6	0.058
HLA-A*69	1	1	0	0	2	0.019
Overall	25	34	13	31	103	1

*NII* non infected individual, *AC* Asymptomatic Carrier, *ATLL* Adult T cell leukemia/lymphoma, *HAM* HTLV-1- associated myelopathy

**Table 4:** Number and frequency of every HLA-B allele found in our population for each group

B	Population				Overall	Allele frequency
	NII	AC	ATLL	HAM		
HLA-B*07	1	2	0	0	3	0.027
HLA-B*08	2	2	0	3	7	0.063
HLA-B*13	0	0	0	1	1	0.009
HLA-B*14	2	2	1	2	7	0.063
HLA-B*15	0	0	0	2	2	0.018
HLA-B*18	0	0	2	0	2	0.018
HLA-B*27	2	0	0	0	2	0.018
HLA-B*35	8	2	3	15	28	0.25
HLA-B*38	4	4	0	0	8	0.071
HLA-B*39	5	4	4	2	15	0.134
HLA-B*40	2	0	0	0	2	0.018
HLA-B*41	0	0	0	1	1	0.009
HLA-B*44	0	2	1	1	4	0.036
HLA-B*48	0	8	0	0	8	0.071
HLA-B*49	0	0	0	1	1	0.009
HLA-B*51	0	3	2	5	10	0.089
HLA-B*52	0	2	0	2	4	0.036
HLA-B*53	0	6	0	0	6	0.054
HLA-B*55	0	0	0	1	1	0.009
Overall	26	37	13	36	112	1

*NII* non infected individual, *AC* Asymptomatic Carrier, *ATLL* Adult T cell leukemia/lymphoma, *HAM* HTLV-1- associated myelopathy

**Table 5:** Number and frequency of every HLA-C allele found in our population for each group

Allele	Population				Overall	Allele frequency
	NII	AC	ATLL	HAM		
HLA-C*01	1	0	0	0	1	0.014
HLA-C*03	10	4	0	4	18	0.243
HLA-C*04	1	6	0	0	7	0.095
HLA-C*05	0	2	0	0	2	0.027
HLA-C*06	0	2	0	0	2	0.027
HLA-C*07	12	7	4	2	25	0.338
HLA-C*12	2	1	0	0	3	0.041
HLA-C*15	0	8	2	0	10	0.135
HLA-C*16	0	3	0	0	3	0.041
HLA-C*18	0	2	0	0	2	0.027
HLA-C*31	1	0	0	0	1	0.014
Overall	27	35	6	6	74	1

*NII* non infected individual, *AC* Asymptomatic Carrier, *ATLL* Adult T cell leukemia/lymphoma, *HAM* HTLV-1- associated myelopathy

In relation to HLA-A, the alleles A*23, A*24, A*32 and A*68 were observed among ATLL patients, but not in HAM/TSP, while the opposite case was found for A*11 and A*29 in HAM/TSP patients but not ATLL. There was a significant difference in the frequency of HLA-A*02 between asymptomatic carriers and those with ATLL (*p* = 0.031) (Table 3, Fig. 2). Allele A*01 was only found among NII; A*25 and A*26, among AC; A*11, among HAM/TSP patients; and A*23 and A*32 among ATLL patients.

For HLA-B, the alleles HLA-B*27 and B*40 were only found in NII; B*48 and B*53, only in AC; B*18, in ATLL; and B*13, B*15, B*41, B*49 and B*55, in HAM/TSP individuals. Nevertheless, these alleles were too rare to draw any conclusions from these findings. The allele B*35 was significantly more frequent among the patients with HAM/TSP compared to asymptomatic carriers (*p* < 0,001) (Table 4, Fig. 2).

**Fig. 1:**
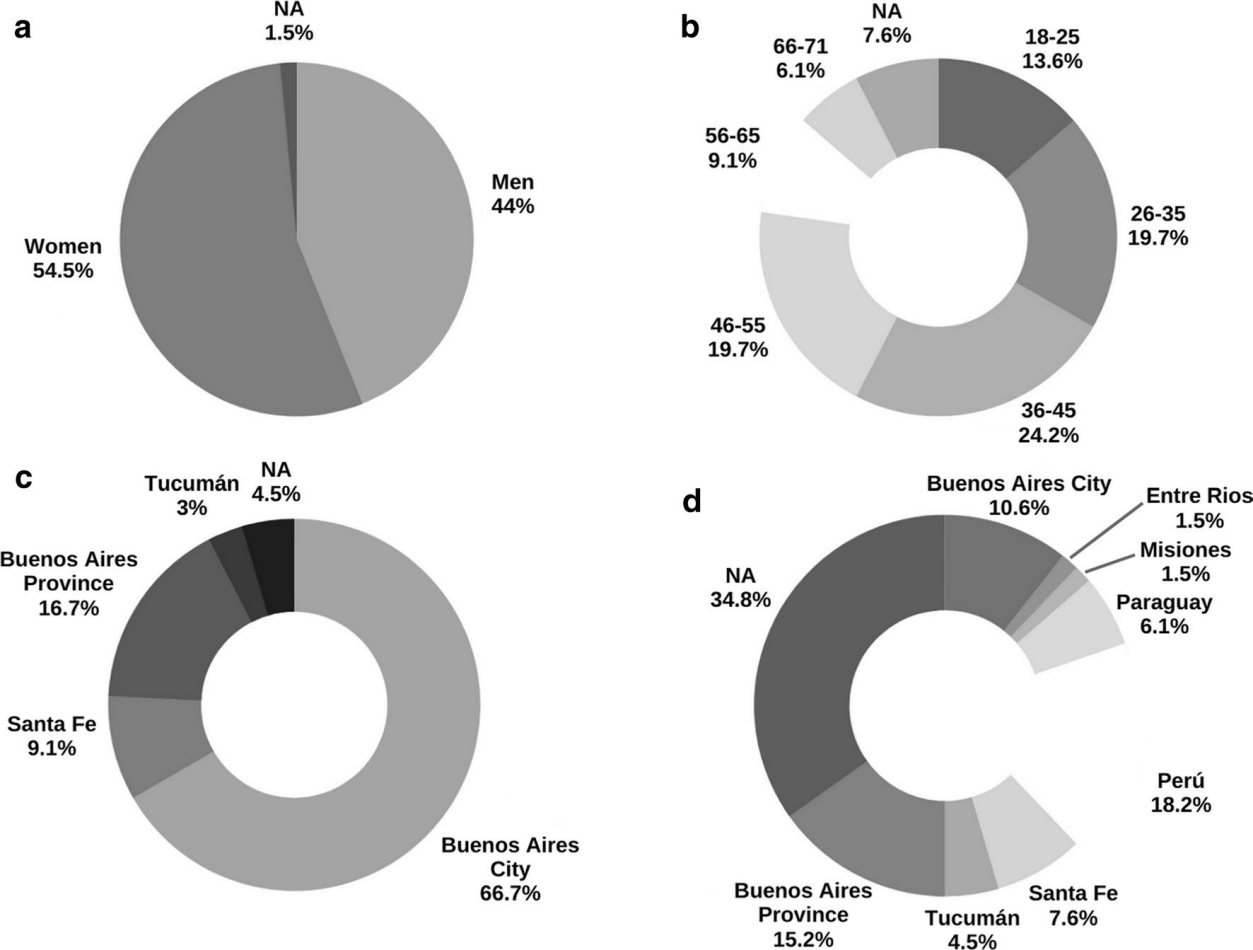
Demographic characteristics of the studied population. **a** Distribution by gender. **b** Distribution by age range. **c** Distribution by region of derivation. **d** Distribution by province/country of birth

**Fig. 2:**
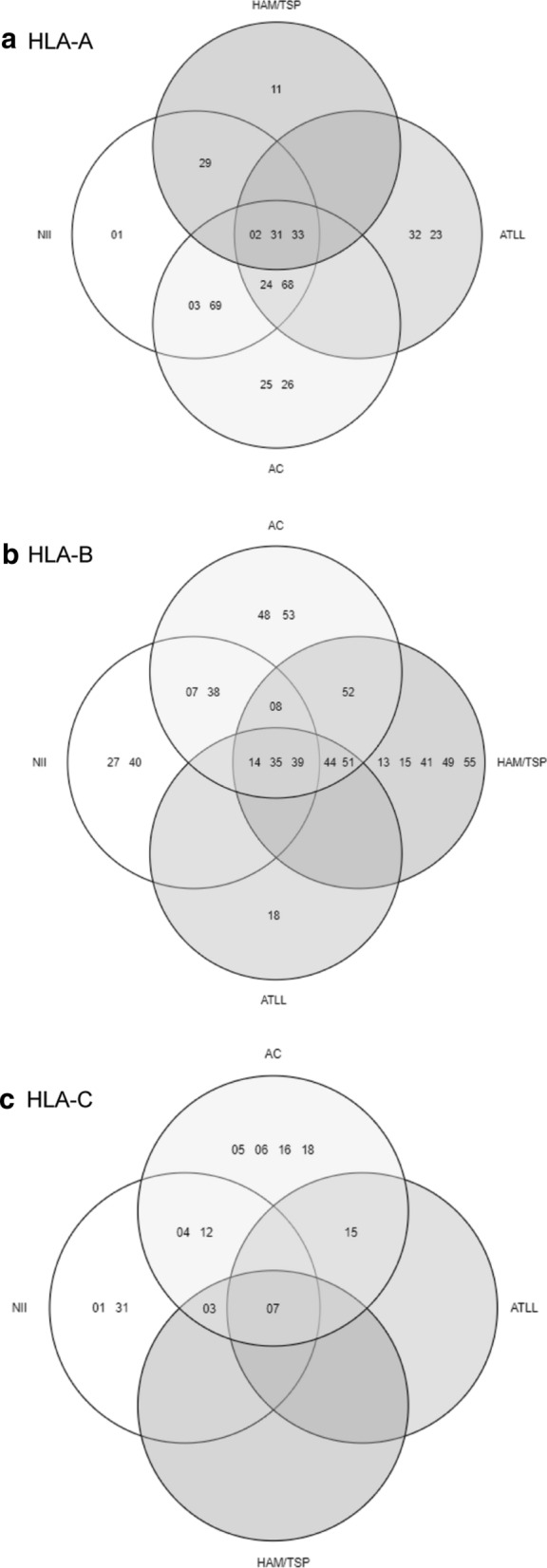
Allele distribution in the different subgroups. **a** Distribution of HLA-A in the 4 subgroups: non-infected individuals (NII), HTLV-1 asymptomatic carriers (AC), patients with Adult T-cell leukemia/ lymphoma (ATLL) and patients with HTLV-1 associated myelopathy/ Tropical Spastic Paraparesis (HAM/TSP). **b** Distribution of HLA-B in the 4 subgroups: NII, AC, ATLL and HAM/TSP. **c** Distribution of HLA-C in the 4 subgroups: NII, AC, ATLL and HAM/TSP

For the 38 HLA-C samples, the alleles C*01 and C*31 were only found in NII, while the alleles C*05, C*06, C*16 and C*18 were exclusive for asymptomatic carriers. There were not any ATLL- or HAM-exclusive alleles. Overall, patients that had developed either one of the associated pathologies exhibited a rather limited arrange of alleles: only C*03, C*07 and C*15 were identified in our analysis. Out of the 3 pathology cases (1 HAM/TSP and 2 ATLL) that presented C*07, all of them were homozygotes for that loci and none displayed the allele A*02. In the AC group, 4 individuals presented C*07 (3 in homocigosis), and one individual was homozygous for A*02 (Fig. 1c, Table 5, Fig. 2). The allele HLA-C*07 was significantly higher among ATLL individuals (*p* = 0.017) compared to AC, but not among HAM/TSP patients (*p* = 0.466).

We repeated the analysis taking in consideration the Argentine individuals only (n = 50), the results showed differences in HLA-A*02, being significantly more frequent in AC than in ATLL (*p* = 0.025); and in HLA-C*07 which did not exhibit any difference among the studied groups. HLA-B*35 was not found in AC, whereas it was present in 12 out of 33 alleles for HAM/TSP and 3 out of 13 for ATLL.

The mean PVL in asymptomatic carriers was 2.78 per 100 PBMCs, and 10.44 in individuals with HTLV-1 associated pathologies (16.36 for ATLL and 8.56 for HAM/TSP patients). PVL of patients with HTLV-1 associated pathologies was significantly higher than that of asymptomatic carriers (*p* = 0.008, Tables 6, 7 and 8) while there were no differences between the mean PVL of ATLL and HAM/TSP patients (*p* = 0.165, Tables 6, 7 and 8). Table 6, 7 and 8 presents the values of PVL classified per HLA-A, HLA-B and HLA-C alleles. No significant differences were observed for HLA-A, B and C when comparing the PVL among all the alleles of each loci by one-way ANOVA.

**Table 6:** Proviral load for each HLA-A allele and clinical status

Allele	Population			Mean PVL
	AC	ATLL	HAM	
HLA-A*02	1.42	12.49*	4.35	3.13
HLA-A*03	4.61*	–	–	4.61
HLA-A*11	–	–	4.03*	4.03
HLA-A*23	–	0.34*	–	0.34
HLA-A*24	6.64	0.34*	–	4.54
HLA-A*25	1.29*	–	–	1.29
HLA-A*26	1.29*	–	–	1.29
HLA-A*29	–	–	9.32**	9.32
HLA-A*31	1.29	33.98	15.1	12.28
HLA-A*32	–	9.82*	–	9.82
HLA-A*33	1.85**	40.2*	11.12	12.88
HLA-A*68	4.61*	1.3*	–	2.95
HLA-A*69	ND	–	–	ND

*AC* asymptomatics carriers, *HAM* HTLV-1 associated myelopathy, *ATLL* Adult T cell leukemia/lymphoma, *NII* Non-infected Individuals, *HLA* Human leukocyte antigen, *PVL* proviral load

^*^Only the PVL of one allele was available, **Mean PVL of two alleles

**Table 7:** Proviral load for each HLA-B allele and clinical status

Allele	Population			Mean PVL
	AC	ATLL	HAM	
HLA-B*07	11.83*	–	–	11.83
HLA-B*08	0.39*	–	15.12	10.21
HLA-B*13	–	–	35.1*	35.1
HLA-B*14	5.3*	1.3*	3.58**	3.44
HLA-B*15	–	–	3.07**	3.07
HLA-B*18	–	0.82**	–	0.81
HLA-B*35	–	5.1**	6.44	6.12
HLA-B*38	2.1**	–	–	2.1
HLA-B*39	0.24	18.78	7.84	10.36
HLA-B*41	–	–	ND	ND
HLA-B*44	0.58*	33.98*	29.54*	21.36
HLA-B*48	1.24	–	–	1.24
HLA-B*49	–	–	35.1*	35.1
HLA-B*51	0.74	12.49*	3.7	4.11
HLA-B*52	0.57*	–	9.32**	6.41
HLA-B*53	3.42	–	–	3.42
HLA-B*55	–	–	4.03*	4.03

*AC* asymptomatics carriers, *HAM* HTLV-1 associated myelopathy, *ATLL* Adult T cell leukemia/lymphoma, *NII* Non-infected Individuals, *HLA* Human leukocyte antigen, *PVL* proviral load

^*^Only the PVL of one allele was available, **Mean PVL of two alleles

**Table 8:** Proviral load for each HLA-C allele and clinical status

Allele	Population			Mean PVL
	AC	ATLL	HAM	
HLA-C*03	2.7**	–	1.675**	2.19
HLA-C*04	0.47	–	–	0.47
HLA-C*05	0.11*	–	–	0.11
HLA-C*06	0.92*	–	–	0.92
HLA-C*07	1.69	40.2*	4.03*	8.5
HLA-C*12	0.58*	–	–	0.58
HLA-C*15	1.89	9.87*	–	3.49
HLA-C*16	2.94**	–	–	2.94
HLA-C*18	0.39*	–	–	0.39

*AC* asymptomatics carriers, *HAM* HTLV-1 associated myelopathy, *ATLL* Adult T cell leukemia/lymphoma, *NII* Non-infected Individuals, *HLA* Human leukocyte antigen, *PVL* proviral load

^*^Only the PVL of one allele was available, **Mean PVL of two alleles

Most of the ATLL and HAM/TSP patients were homozygous for HLA-A (20/21) locus, in contrast to the HLA-B locus (4/21). Regarding heterozygosity, for the HLA-A locus there was a significant difference when comparing asymptomatics versus individuals with pathologies, heterozygosity being more frequent among asymptomatics (*p* = 0.038, Table 9). Nevertheless, all of the individuals with ATLL were heterozygous for HLA-B.

**Table 9:** Number of homozygous patients for each gene

Population	HLA-A	HLA-B	HLA-C
NII	10/12	11/12	11/13
AC	12/17	18/18	15/17
ATLL	5/6	0/6	3/3
HAM	15/15	4/15	3/3
Overall	42/50	33/51	32/36

We found all of the ATLL and HAM patients that could be typed for HLA-C to be homozygous for this gene and all of the asymptomatic carriers to be homozygous for HLA-B (Table 9).

## Discussion

It has been proposed that the HTLVs have arisen as a consequence of inter-species transmissions that took place millions of years ago in Africa and that HTLV-1 was introduced in America during the multiple pre-Columbian Mongoloid migrations over the Bering Strait, and in the post-Columbian era from Japan and with the slave trade from Africa. Therefore, the different migration waves of infected populations resulted in an ethnic/geographical restriction in the American continent for HTLV-1 and 2. Moreover, Lou et al*.* described certain polymorphisms as possibly associated to susceptibility for HTLV-1 infection for ethnically related populations of Russia (HLA-A*02, A*24) and Japan (HLA-A*24 and A*26) [15, 34]. In the general population of Argentina the reported frequencies of A*02, A*24 and A*26 were 24.95%, 11.25% and 4.02%, respectively [35]. In the studied population (Table 3, 4 and 5) there was a high frequency of allele A*02 (36.89%), and following the tendency of the data reported in the general population, the frequencies of A*24 (8.74%), A*26 (0.97%) were lower. When comparing the group of NII against HTLV-1^+^ individuals, there were no significant differences between them, therefore a bigger sample size could determine if there is an association regarding their frequency and HTLV-1 infection status. In the case of HLA-B and -C, the most common alleles in Argentina are B*35 (14.6%), B*44 (11.4%) and B*51 (7.9%), and C*07 (24.6%), C*04 (16.6%) and C*03 (10.4%) (35). Our own population largely matched these data, except for the cases of alleles B*44 and C*04, which were relatively uncommon (3.6% and 9.5%, respectively). B*44 was only present in HTLV-1 individuals while for C*04 no significant differences were found when comparing non-infected individuals vs HTLV-1 patients (p = 0.097). Instead, alleles B*39 (13.4%), C*03 (24.3%) and C*15 (13.5%) were among the top found. It should be noted that all the alleles are present in a lower proportion in the general population than in our own due to the fact that there was a smaller variety of alleles found in the latter, which translates in a bigger proportion of the total distribution for each of them.

Regarding, the thirteen Peruvian and the four Paraguayan individuals sampled, the allele frequencies matched the reported prevalence in these populations [36, 37]. The most frequent alleles for HLA-A in the general population in Peru were A*02, A*24 and A*68, in decreasing order, which correlated with our own findings. The same happened for HLA-B (most frequent HLA-B*35), and HLA-C, the most common allele being Cw*04. In our own population, we found 7 copies of said allele, 4 of which corresponded to Peruvian individuals. When it came to the four Paraguayan individuals tested, the most common alleles were HLA-A*02 and HLA-B*35, the same as for the general population in that country, although for the case of the indigenous Guaraní, the most common HLA-B alleles were HLA-B*15 and B*40. Nevertheless, when repeating the analysis taking in consideration the Argentine individuals only (n = 50), for HLA-A*02 the difference between AC and ATLL is still conserved (*p* = 0.025) in concordance to reports that associated this allele to protection against developing HTLV-1 associated pathologies. For HLA-B*35, we found no AC individuals that carried the allele, which indicates an even stronger correlation between this allele and susceptibility to HAM/TSP. HLA-C*07, nonetheless, was no longer correlated to susceptibility to ATLL when excluding non-Argentine individuals.

Regarding the pathologies associated to HTLV-1 infection, it is known that most individuals remain asymptomatic throughout their lives. ATLL and HAM/TSP patients are more frequent in areas of high endemicity and they represent a small percentage of the infected population (up to 5%).

The reasons behind the development of pathologies during adulthood and their association to host genetic factors are still unclear even though many hypotheses have been proposed. HLA class I genes may have an effect on the progression towards ATLL and HAM/TSP due to its critical role in antigen presentation [15]. Various alleles have been described as either protective or susceptible for the development of ATLL or HAM/TSP. In Jamaica, Japan and Brazil the allele HLA-A*02 has been described as protective both for ATLL and HAM/TSP, in accordance with other studies which reported finding it significantly more frequent in asymptomatic carriers [15, 19, 34]. In our population, A*02 was significantly rarer in ATLL patients when compared to HTLV-1^+^ asymptomatic carriers, suggesting as well a protective role for this allele in this group. This protective role could not be confirmed for HAM/TSP patients in this study. The allele HLA-A*03, previously described as protective in Jamaica, was only found in asymptomatic patients and healthy donors in the studied population and with a low frequency.

We did not found any associations linking HLA-A*26 to either protection or susceptibility to ATLL, although it should be pointed out that this allele is not frequently found in the Argentine population (4.02%) [35] and it was even rarer in our population, having been found only in one individual.

In the case of HLA-B allele distribution, B*35 was found to be significantly more frequent in patients with HAM/TSP than in asymptomatics, which could point to a possible association of this allele to the development of diseases. Although B*35 has been previously linked to progression to disease, viral load, heterosexual transmission and mother to child transmission in HIV-1 infected individuals and to disease progression in HBV, this is the first report about this allele in relation to HTLV-1 infection [38–40].

Regarding HLA-C, our analysis yielded similar results with previous studies in Brazil and Japan, which reported a correlation between HLA-C*07 and progression to disease, even though in our case it was associated to ATLL (*p* = 0.017) and not to HAM/TSP (p = 0.466) [16, 25]. None of the individuals with pathologies presented HLA-C*07 and HLA-A*02 concomitantly, and only one out of four AC had them both; therefore, we could not find the protective effect of HLA-A*02 from HLA-C*07.

Another aspect to be considered is that all of the alleles found solely in one group had a very low frequency; many of them were actually identified that one time (Table 2). They were also very rare alleles for the general Argentine population [35]. Thus, it is not possible to draw any conclusions regarding these findings. The only exceptions were HLA-B*48 and HLA-B*53, which had a frequency of 7.14% (8/112) and 5.36% (6/112) (Table 4), respectively and were found solely in AC.

Many biomarkers have been proposed as prognostic for development of either ATLL (PD-1/PD-L1, absence of CD7 in CD4^+^ T cells [41–43] or HAM (CXCL10, CXCL9, neopterin and HTLV-1 antibody titers in CSF, and gender [44–46], although the most studied is PVL.

To this day, the therapies for HTLV-1 associated pathologies seek to reduce the proviral load. In the last decade, a real time quantitative PCR (qPCR) has been implemented for the quantification of proviral load (PVL) of HTLV-1/2 from cells of infected patients. Its determination is used as an indicator of the course of infection in asymptomatic carries in order to evaluate their predisposition to the development of pathology and to monitor treatment progression in ATLL and HAM patients [47]. It has been reported that, although the PVL has been suggested to be directly related to the severity of the disease, the values among infected individuals often vary significantly [48]. This corresponds with the dispersion of the values observed in this study. Previously reported values indicate that in asymptomatic carriers, the mean proviral load is 0.1–1 copy/100 PBMCs, while in patients with HAM/TSP is 5–10/100 PBMCs, exceeding sometimes 30 copies [48]. Despite these differences observed in the PVL values and the technique used, all the reports conclude that there is a significant difference among asymptomatics and patients with pathologies as observed in our studied population. These results also indicate that there is a correlation towards disease progression [33].

Some studies have proposed that HLA allelic variants could determine the PVL levels of HTLV-1 infected individuals [14, 49]. Nonetheless, we couldn't find any significant differences in the PVL of any allele to support these previous claims (Tables 6, 7 and 8).

It has been proposed that heterozygosis on HLA confers advantages on disease progression in AIDS, revealing a greater variety of the immune response [50, 51]. In accordance to this, heterozygosis for HLA-A was significantly more frequent among asymptomatics when compared to individuals with pathologies. However, the opposite was true for HLA-B, for which homozygosis was more frequent in asymptomatic carriers than in patients with pathologies (Table 9).

In conclusion, several HLA alleles identified in our study were associated with disease progression. Our results add more evidence to the protective effect of HLA-A*02 allele on progression to ATLL, and draws attention to HLA-B*35 as a new allele to be considered in relation to susceptibility to HAM/TSP, and also HLA-C*07 in relation to progression to ATLL.

To this day, however, no allele or allele pattern has been identified to be exclusive to either asymptomatic individuals or those who develop pathologies, and to thus be of use when it comes to providing a predictive diagnosis. Were an allele like this to be found, in line with the rapidly evolving field of precision medicine, it would mean the possibility to conduct a closer follow up of each asymptomatic HTLV-1^+^ carrier, for those patients that choose to learn the impact of their genetic background on the infection by HTLV-1.

## Conclusions

We have found HLA-A*02, HLA-B*35 and HLA-C*07 to be associated to protection from ATLL (HLA-A*02) and susceptibility to HAM/TSP (HLA-B*35) or to ATLL (HLA-C*07), respectively. Whereas HLA-A*02 protection from ATLL has already been extensively described in other regions of the world, this is the first report that links HLA-B*35 and an increased susceptibility to HAM/TSP. As for HLA-C*07 it has previously been associated to susceptibility to HAM/TSP in other countries but in our population it has been linked to ATLL.

These alleles could be of relevance, among other markers, to determine a model for disease development prognosis and helping the generation of a vaccine for use in different geographical areas around the world.

## Data Availability

The datasets used and/or analyzed during the current study are available from the corresponding author on reasonable request.

## Abbreviations

Ac: Asymptomatic carriers
AIDS: Acquired Immunodeficiency Syndrome
ANOVA: Analysis of Variance
CONICET: Consejo Nacional de Investigaciones Científicas y Técnicas
CNS: Central Nervous System
CTL: Cytotoxic T Lymphocyte
EDTA: Ethylenediaminetetraacetic Acid
ELISA: Enzyme-Linked Immunosorbent Assay
EM: Expectation–Maximization
DNA: Desoxyribonucleic Acid
HAM/TSP: HTLV-1 Associated Myelopathy/Tropical Spastic Paraparesis
HBV: Hepatitis B Virus
HBZ: HTLV-1 basic leucine zipper factor
HIV-1: Human Immunodeficiency Virus type 1
HLA: Human Leukocyte Antigen
HTLV-1: Human T lymphotropic virus type 1
IFNγ: Interferon gamma
ITAPS: International Traineeships in AIDS Prevention Studies
n-PCR: Nested Polymerase Chain Reaction
NIH: National Institute of Health
NII: Non-Infected Individuals
PVL: Proviral Load
PBMC: Peripheral Blood Mononuclear Cells
SBT: Sequence-Based Typing
TNF: Tumor Necrosis Factor
